# The Prognostic Role of Vimentin Expression in Triple‐Negative Breast Cancer: A Retrospective Cohort Study

**DOI:** 10.1002/cnr2.70632

**Published:** 2026-10-13

**Authors:** Erwin Danil Yulian, Tantri Hellyanti, Karl Heinz Leonhardt Rowika

**Affiliations:** ^1^ Division of Surgical Oncology, Department of Surgery, Faculty of Medicine Universitas Indonesia Jakarta Indonesia; ^2^ Department of Anatomical Pathology, Faculty of Medicine Universitas Indonesia Jakarta Indonesia; ^3^ Department of Surgery, Faculty of Medicine Universitas Indonesia Jakarta Indonesia

## Abstract

**Introduction:**

Triple‐negative breast cancer (TNBC) is a subtype of breast cancer with the poorest prognosis and aggressive biological behavior. In Indonesia, approximately 15%–25% of breast cancer cases fall into the TNBC category. One of the biological markers involved in the epithelial–mesenchymal transition (EMT) process and tumor progression of TNBC is vimentin. Positive vimentin expression has been reported to correlate with reduced survival; however, data in the Indonesian population remain limited.

**Methods:**

This retrospective study included 29 TNBC patients who received treatment at Dr. Cipto Mangunkusumo National General Hospital between 2017 and 2022, with a 3‐year follow‐up period. Clinicopathological data and vimentin expression were analyzed using bivariate analysis and Kaplan–Meier survival analysis.

**Results:**

Most patients were aged ≥ 40 years (82.8%), had a normal body mass index (82.8%), presented with locally advanced disease (72.4%), high histopathological grade (62.1%), and high Ki‐67 expression (72.4%). A significant association was observed between vimentin expression and overall survival (*p* = 0.019), where increased vimentin expression was associated with reduced survival (mean survival 30.42 months in the negative group vs. 20.86 months in the positive group). Vimentin expression was also significantly associated with Ki‐67 expression (*p* = 0.045), indicating a link between cellular proliferation and activation of EMT. Further analysis in patients with TNBC demonstrated that positive vimentin expression significantly increased the risk of mortality (*p* = 0.015; HR 5.64; 95% CI 1.39–22.7), while inadequate treatment was also associated with poorer overall survival (*p* = 0.011; HR 4.05; 95% CI 1.37–11.9).

**Conclusion:**

Vimentin expression is significantly associated with reduced overall survival in patients with TNBC and correlates with high Ki‐67 expression, reflecting tumor aggressiveness and activation of EMT. Inadequate treatment also contributes to poorer survival outcomes. These findings highlight the potential role of vimentin as a prognostic molecular marker in TNBC within the Indonesian population.

## 1 Introduction

Breast cancer remains the most common malignancy and a leading cause of cancer‐related death among women worldwide. According to GLOBOCAN 2020, breast cancer accounts for approximately 2.3 million new cases and 685 000 deaths annually [1]. In Indonesia, breast cancer is also the most prevalent malignancy, with 68 690 new cases reported in 2022 [2].

Among subtypes, triple‐negative breast cancer (TNBC) accounts for 15%–25% of all cases and is characterized by the absence of estrogen receptor (ER), progesterone receptor (PR), and HER2 expression [3]. TNBC exhibits aggressive clinical behavior, early metastasis, and limited therapeutic options, leading to poor outcomes compared with other breast cancer types [4, 5].

The epithelial–mesenchymal transition (EMT) plays a central role in TNBC progression by promoting migration, invasion, and treatment resistance [6]. Vimentin, a mesenchymal intermediate filament protein, is a hallmark of EMT and has been associated with tumor invasiveness and poor prognosis in several malignancies [7, 8, 9, 10]. However, evidence from Indonesian TNBC populations remains limited.

This study aimed to evaluate the association between vimentin expression and overall survival (OS) among Indonesian TNBC patients and to assess its relationship with clinicopathological characteristics.

## 2 Methods

### 2.1 Study Design and Population

This retrospective cohort study included female TNBC patients treated at Dr. Cipto Mangunkusumo National General Hospital (RSCM) between 2017 and 2022. Inclusion criteria were histologically confirmed TNBC (ER ≤ 1%, PR ≤ 1%, HER2 0–1+ or 2+ with negative ISH), availability of paraffin blocks for immunohistochemistry (IHC), and complete clinical records. A total of 29 patients met the criteria.

### 2.2 Data Collection and Variables

Data collected included age, body mass index (BMI), tumor size, lymph node status, histological grade, clinical stage, Ki‐67 expression, treatment modality, and OS. Vimentin expression was assessed by IHC and categorized as negative (0) or positive (1+ to 3+). For the evaluation of vimentin expression, only cytoplasmic staining in invasive tumor epithelial cells was scored, whereas staining observed in stromal fibroblasts, endothelial cells, inflammatory cells, and extracellular debris was excluded from the analysis. Stromal staining served as an internal positive control and was not included in the scoring assessment. Vimentin‐stained slides were independently evaluated by the investigator and a consultant anatomical pathologist. In cases of discordant interpretation, the slides were reviewed jointly to reach a consensus, with the consultant anatomical pathologist providing the final determination. OS was defined as the time from diagnosis to death from any cause or last follow‐up.

Archived formalin‐fixed paraffin‐embedded (FFPE) tissue blocks obtained from breast biopsy or surgical specimens were used for immunohistochemical analysis. Tissue sections (3 μm thick) were prepared and stained using a standardized IHC protocol. Briefly, following deparaffinization and rehydration, antigen retrieval was performed using Tris‐EDTA buffer (pH 9.0), followed by blocking procedures. The sections were then incubated with anti‐vimentin primary antibody (Novocastra; dilution 1:100) for 1 h. Immunoreactivity was visualized using diaminobenzidine (DAB) chromogen and counterstained with hematoxylin before dehydration, clearing, and mounting.

### 2.3 Statistical Analysis

Data were analyzed using SPSS version 20.0. Associations between vimentin expression and clinicopathological variables were evaluated using chi‐squared or Fisher's exact tests. Survival curves were generated using the Kaplan–Meier method, and differences were assessed using the log‐rank test. A *p*‐value < 0.05 was considered statistically significant.

## 3 Results

### 3.1 Patient Characteristics

The clinicopathological characteristics of the study population are summarized in Table 1. A total of 29 female patients with TNBC treated between 2017 and 2022 were included. The majority were aged ≥ 40 years (82.8%), and most had a normal BMI (82.8%). Clinically, 72.4% of patients presented with locally advanced disease, while 62.1% had high histological grade (Grade III) tumors. A high Ki‐67 index (≥ 20%) was observed in 72.4% of cases, indicating elevated proliferative activity.

**TABLE 1:** Clinicopathological characteristics of TNBC patients (*n* = 29).

Variable	*n*	%
Age		
< 40 years old	5	17.2
≥ 40 years old	24	82.8
Body mass index (BMI)		
< 25	24	82.8
≥ 25	5	17.2
Tumor size		
T1–T2	9	31
T3–T4	20	69
Lymph node status		
N0	7	24.1
N1–N3	22	75.9
Clinical stage		
Early	3	10.3
Locally advanced	26	89.7
Adequate therapy		
Yes	21	72.4
No	8	27.6
Ki67 expression		
High (≥ 20%)	21	72.4
Low (< 20%)	8	27.6
Histopathological grade		
Grade I–II	11	37.9
Grade III	18	62.1
Lymphovascular invasion		
Yes	4	13.8
No	25	86.2
Histopathological type		
Ductal invasive/NST	28	96.6
Mixed invasive ductal and lobular carcinoma	1	3.4
Mastectomy		
Yes	25	86.2
No	4	13.8
Chemotherapy		
Yes	23	79.3
No	6	20.7
Radiotherapy		
Yes	10	69
No	9	31
Outcome		
Event	22	75.9
Censored	7	24.1

### 3.2 Vimentin Expression

The distribution of vimentin expression in tumor tissues is presented in Table 2. Vimentin expression was assessed semiquantitatively based on staining intensity in tumor cells. The staining score was categorized as follows: Score 0, negative (no positive staining observed in tumor cells); Score 1+, weak cytoplasmic staining intensity in > 10% of tumor cells; Score 2+, moderate cytoplasmic staining intensity in > 10% of tumor cells; and Score 3+, strong cytoplasmic staining intensity in > 10% of tumor cells. Cytoplasmic staining was considered positive. Vimentin expression was detected in 22 of 29 cases (75.9%). Among these, 2 cases (7.0%) showed weak (1+) expression, 6 (20.7%) moderate (2+), and 14 (48.3%) strong (3+) expression. Representative immunohistochemical staining of vimentin is shown in Figure 1.

**TABLE 2:** Vimentin expression in tumor tissue.

Vimentin expression	Total	
	*n*	%
Vimentin expression level (score)		
0	7	24.1
1+	2	7.0
2+	6	20.7
3+	14	48.3

Vimentin expression category		
Positive (Score 1+, 2+, 3+)	22	75.9
Negative (Score 0)	7	24.1

**FIGURE 1:**
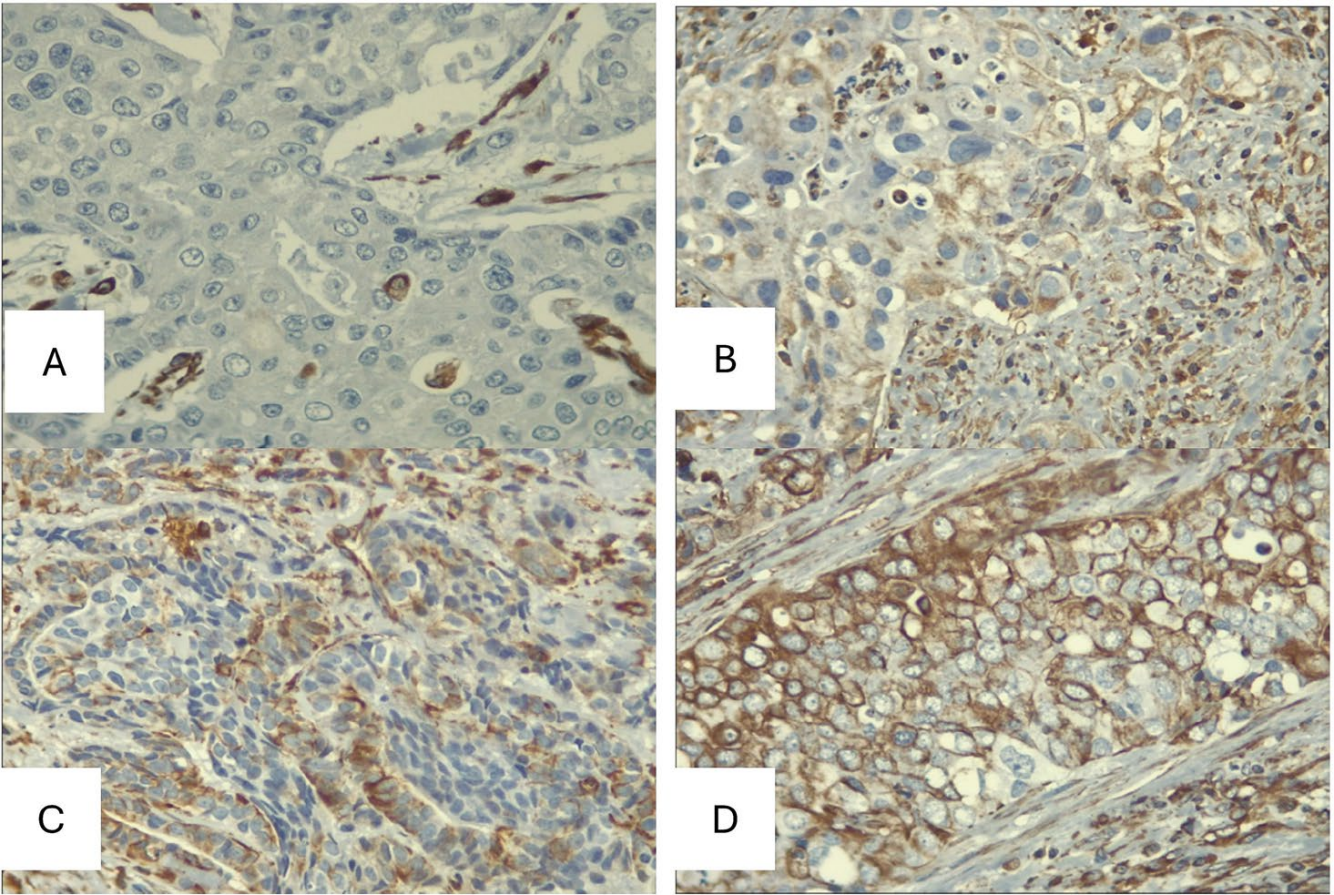
Representative immunohistochemical staining of vimentin. (A) Negative vimentin expression, Score 0; (B) Positive vimentin expression, Score 1+; (C) Positive vimentin expression, Score 2+; (D) Positive vimentin expression, Score 3+.

### 3.3 Association Between Vimentin Expression and OS

The correlation between vimentin expression and OS in patients with TNBC is presented in Table 3. Statistical analysis revealed a significant association between vimentin expression and OS (*p* = 0.019). Among the 22 patients exhibiting positive vimentin expression, 19 experienced mortality events, while 3 remained alive at the end of the observation period. In contrast, among the seven patients with negative vimentin expression, only three experienced mortality events and four were alive at the final follow‐up.

**TABLE 3:** Association between vimentin expression and overall survival.

Variable	Overall survival		Nilai P
	Event	Sensor	
Vimentin			**0.019**
Positive	19	3	
Negative	3	4	

The Kaplan–Meier survival curves illustrating differences in OS according to vimentin expression status are shown in Figure 2. Patients with positive vimentin expression had a shorter mean survival time of 20.86 ± 2.01 months (95% CI 16.90–24.82), whereas those with negative expression demonstrated a longer mean survival of 30.42 ± 4.36 months (95% CI 21.87–38.98). The overall mean survival of all patients was 23.27 ± 2.03 months (95% CI 19.28–27.26).

**FIGURE 2:**
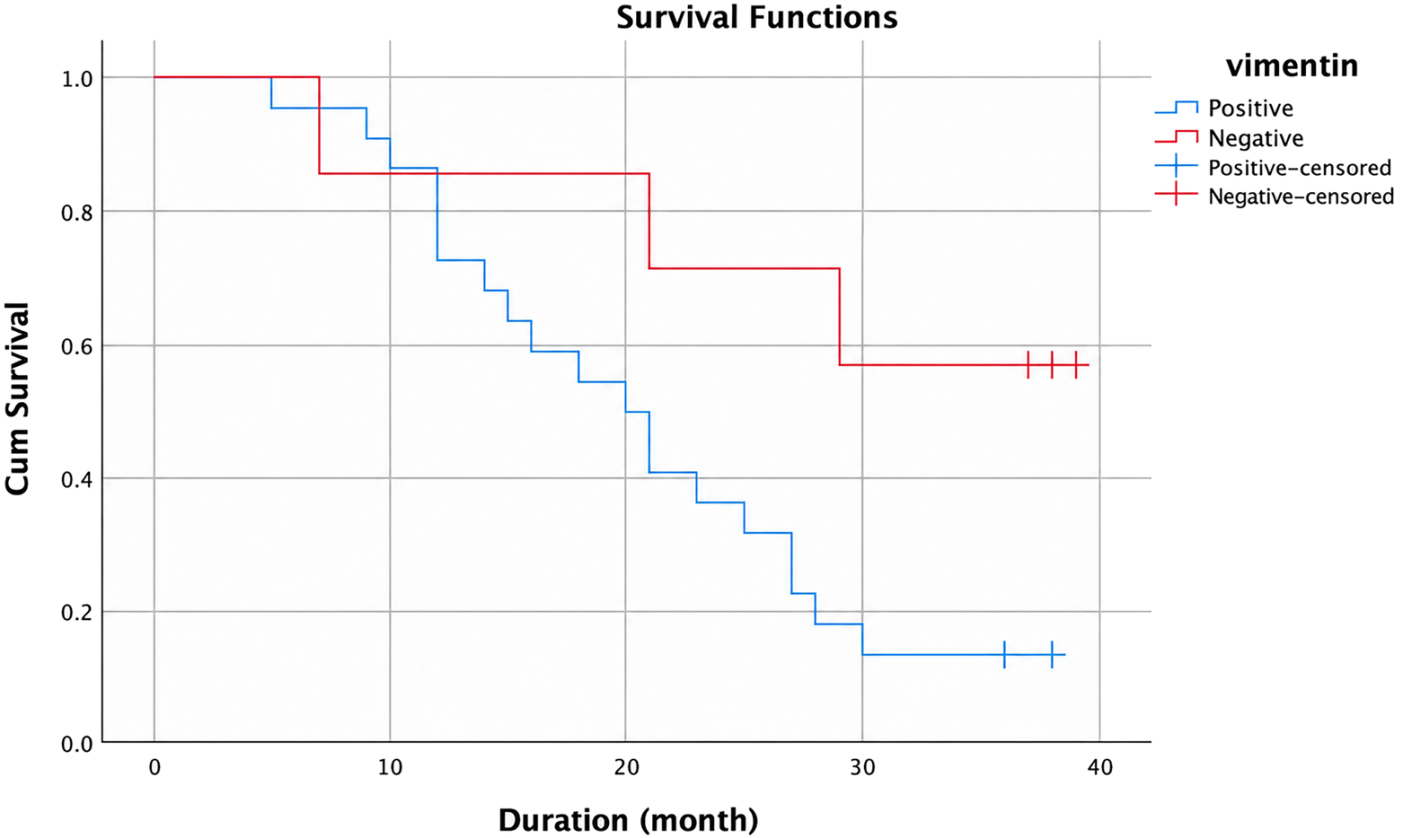
Kaplan–Meier overall survival curve of samples based on vimentin expression.

The log‐rank test revealed a significant difference between the two survival curves (*p* = 0.004), indicating that vimentin expression status significantly affects patient prognosis.

### 3.4 Correlation Between Vimentin and Clinicopathological Factors

The association between vimentin expression and clinicopathological characteristics is presented in Table 4. No significant associations were observed between vimentin expression and conventional clinicopathological parameters, including age, BMI, tumor size, lymph node status, and clinical stage (all *p* > 0.05). However, vimentin expression showed a significant correlation with Ki‐67 expression (*p* = 0.045), suggesting a link between EMT activation and proliferative activity.

**TABLE 4:** Correlation between vimentin expression and clinicopathological characteristics.

Variable	*p* (chi‐square)
Age	0.563
Body mass index (BMI)	0.563
Tumor size	0.361
Lymph node status	0.146
Histopathological grade	0.387
Ki67 expression	**0.045**
Adequate therapy	0.062

### 3.5 Correlation Between Clinicopathological Factors and Survival

The association between clinicopathological variables and OS according to Cox regression analysis is presented in Table 5. None of the conventional clinicopathological variables, including tumor size, lymph node status, histopathological grade, and Ki‐67 expression, showed a statistically significant association with OS (all *p* > 0.05). In contrast, vimentin expression and adequate therapy were significantly associated with OS in the Cox regression analysis, with hazard ratios (HR) of 5.64 (95% CI: 1.39–22.7; *p* = 0.015) and 4.05 (95% CI: 1.37–11.9; *p* = 0.011), respectively.

**TABLE 5:** Association between clinicopathological variables and overall survival.

Variable	*p* value (Cox‐regression)	HR (95% CI)
Tumor size	0.337	0.62 (0.23–1.64)
Lymph node status	0.964	1.02 (0.33–3.13)
Histopathological grade	0.337	0.62 (0.23–1.64)
Ki67 expression	0.505	0.67 (0.21–2.14)
Vimentin	**0.015**	**5.64 (1.39–22.7)**
Adequate therapy	**0.011**	**4.05 (1.37–11.9)**

## 4 Discussion

### 4.1 Patient Characteristics

Most patients in this study were aged ≥ 40 years (82.8%), indicating that TNBC in Indonesia tends to occur in older women. While TNBC is classically described as affecting younger patients, regional studies in Southeast Asia show a similar age shift, likely reflecting genetic and environmental differences [11, 12]. The lower prevalence of *BRCA1* mutations in Asian populations compared with Western cohorts suggests that nongenetic factors, such as hormonal imbalance and oxidative stress, may contribute more significantly to TNBC pathogenesis [13].

The predominance of patients with normal BMI (< 25; 82.8%) suggests that obesity was not a major risk factor in this cohort. However, normal BMI may mask visceral adiposity, which can still promote tumorigenesis through inflammatory cytokines (TNF‐α, IL‐6) that activate NF‐κB signaling and angiogenesis [14].

Late‐stage presentation (72.4% locally advanced) reflects persistent barriers in early breast cancer detection in Indonesia, including low health literacy and limited access to diagnostic services [15]. This contributes directly to poorer survival outcomes, as advanced TNBC typically has a 5‐year survival below 50%.

Histopathologically, most tumors were Grade III with high Ki‐67 expression, consistent with basal‐like TNBC biology. High Ki‐67 indicates active proliferation and correlates with PI3K–AKT–mTOR pathway activation, which promotes cell‐cycle progression and inhibits apoptosis [16, 17]. These characteristics explain the aggressive nature and early recurrence pattern of TNBC, particularly within the first 2–3 years after therapy.

Overall, these findings depict the clinical and biological landscape of TNBC in developing countries, delayed diagnosis, limited targeted therapy, and predominance of aggressive basal‐like phenotypes, emphasizing the importance of biomarker‐driven prognostication.

### 4.2 Vimentin Expression and OS

Vimentin expression showed a significant association with OS (*p* = 0.019). Mean survival decreased from 30.42 ± 4.36 months in vimentin‐negative patients to 20.86 ± 2.01 months in those with strong (3+) expression. This supports vimentin as an indicator of poor prognosis in TNBC.

Vimentin, a mesenchymal intermediate filament protein, plays a central role in EMT, a process that enhances cancer cell motility, invasiveness, and metastatic potential [18]. Activation of EMT is regulated by transcription factors such as Snail, Twist, and Slug, as well as signaling pathways including TGF‐β/SMAD, STAT3, and Wnt/β‐catenin [19, 20]. Overexpression of vimentin reflects EMT activation, cytoskeletal remodeling, and increased therapeutic resistance. However, because vimentin is also constitutively expressed in normal mesenchymal stromal cells, distinguishing tumor‐cell expression from stromal expression is essential when evaluating its prognostic significance. Therefore, in the present study, only cytoplasmic staining within invasive tumor epithelial cells was assessed, while stromal staining was excluded from the analysis to minimize potential confounding effects.

Previous studies support these findings. Xu et al. [21] reported that vimentin overexpression in TNBC enhances invasion and anthracycline resistance, while Fedele et al. [22] found that high vimentin levels doubled mortality risk and were associated with distant metastasis, especially to the lung and brain. These data suggest that vimentin intensity reflects tumor aggressiveness through EMT‐driven pathways such as Wnt/β‐catenin and PI3K–AKT, which regulate *CCND1* and *MYC* expression and inhibit apoptosis [23].

Clinically, vimentin may serve as a prognostic biomarker and potential therapeutic target. Preclinical studies have shown that inhibitors of TGF‐β and STAT3 can attenuate EMT signaling and tumor invasiveness, offering promise for personalized TNBC therapy.

### 4.3 Correlation Between Vimentin and Clinicopathological Factors

No significant correlations were observed between vimentin expression and conventional clinicopathological factors, except for Ki‐67 (*p* = 0.045). This association implies that proliferation and EMT activation may occur in parallel, as both are regulated by TGF‐β and β‐catenin signaling, which induce *vimentin*, *CCND1*, and *MYC* transcription [21]. Inflammatory cytokines (IL‐6, TNF‐α) further enhance this process via the JAK/STAT pathway, supporting tumor cell survival under oxidative and hypoxic stress.

Patients with locally advanced disease tended to exhibit higher vimentin expression, consistent with its localization at invasive tumor fronts where matrix degradation and stromal interactions are active [18]. Vimentin‐positive cells secrete metalloproteinases (MMP‐2, MMP‐9), facilitating extracellular matrix breakdown and invasion.

The lack of association between vimentin and tumor grade likely reflects sample homogeneity, as most tumors were already high grade. Nevertheless, the predominance of high‐grade, vimentin‐positive tumors reinforces the biological aggressiveness of TNBC.

### 4.4 Clinicopathological Variables and Survival

Cox regression analysis demonstrated that vimentin expression and adequate therapy were significantly associated with OS. In contrast, other clinicopathological variables, including histopathological grade, tumor size, and lymph node status, were not significantly associated with survival outcomes. Consistent with these findings, patients with positive vimentin expression showed a higher risk of mortality compared with other groups, suggesting that biological factors may play a more dominant role than anatomical characteristics in determining prognosis in TNBC [12, 24].

Molecular mechanisms such as EMT activation, *p53* mutation, and PI3K–AKT dysregulation have a stronger impact on survival than tumor size alone [13, 16]. Studies have demonstrated that EMT‐high TNBCs have shorter median survival despite similar clinical staging [13, 19, 24]. Thus, conventional clinicopathological variables may underestimate true biological aggressiveness.

Integrating molecular profiling, especially vimentin expression, into clinical assessment could improve prognostic accuracy and guide targeted therapy. Patients with high vimentin levels may benefit from EMT‐inhibitory strategies, such as TGF‐β or STAT3 blockade, which are being explored in TNBC trials [23]. Incorporating vimentin immunostaining into routine diagnostic panels may therefore enhance risk stratification and personalized management, particularly in tertiary centers such as RSCM.

### 4.5 Study Limitation

Several limitations should be acknowledged. First, the study included a relatively small number of patients from a single institution, which may have limited the statistical power to detect significant associations. Second, most patients presented with locally advanced disease, resulting in a cohort that may not fully represent the broader spectrum of TNBC. Therefore, caution is warranted when generalizing these findings to early‐stage TNBC populations. Future multicenter studies with larger and more heterogeneous cohorts are needed to validate the prognostic significance of vimentin expression.

## 5 Conclusion

Most TNBC patients at Dr. Cipto Mangunkusumo National Referral Hospital were aged ≥ 40 years, presented at advanced stages, and exhibited high histopathological grade with elevated Ki‐67 expression, reflecting an aggressive tumor profile. Vimentin expression was significantly associated with OS (*p* = 0.019), with higher expression linked to poorer outcomes. Ki‐67 expression was also significantly associated with vimentin (*p* = 0.045), suggesting a relationship between increased proliferation and EMT activation. In multivariate analysis, positive vimentin expression (HR 5.64; *p* = 0.015) and adequate therapy (HR 4.05; *p* = 0.011) remained significant predictors of OS.

Future prospective studies with larger sample sizes are needed to validate these findings, and incorporation of vimentin assessment into routine immunohistochemical panels may support risk stratification and individualized treatment planning.

## Author Contributions

E.D.Y. contributed to the study conception and design, methodology development, data analysis and interpretation, drafting of the manuscript, critical revision of the manuscript, and final approval of the version to be published. T.H. contributed to the study design, immunohistochemical evaluation and interpretation of vimentin‐stained slides, data interpretation, critical revision of the manuscript, and final approval of the version to be published. K.H.L.R. contributed to the study conception, methodology development, data collection and interpretation, statistical analysis, drafting and substantial revision of the manuscript, supervision of the study, and final approval of the version to be published. All authors have read and approved the final manuscript and agree to be accountable for all aspects of the work.

## Funding

The authors have nothing to report.

## Ethics Statement

This study was approved by the Ethics Committee of Dr. Cipto Mangunkusumo General Hospital, Faculty of Medicine, Universitas Indonesia.

## Consent

The requirement for informed consent was waived due to the retrospective nature of the study and the use of anonymized archival tissue samples.

## Conflicts of Interest

The authors declare no conflicts of interest.

## Data Availability

The data that support the findings of this study are available from the corresponding author upon reasonable request.

## References

[1] H. Sung , J. Ferlay , R. L. Siegel , et al., “Global Cancer Statistics 2020: GLOBOCAN Estimates of Incidence and Mortality Worldwide for 36 Cancers in 185 Countries,” CA: A Cancer Journal for Clinicians 71, no. 3 (2021): 209–249. 10.3322/caac.21660 33538338

[2] Z. Wang , L. Liu , Y. Li , et al., “Analysis of CK5/6 and EGFR and Its Effect on Prognosis of Triple Negative Breast Cancer,” Frontiers in Oncology 10 (2021): 575317. 10.3389/fonc.2020.575317 PMC7855982 33552956

[3] A. C. Wolff , M. E. H. Hammond , D. G. Hicks , et al., “Recommendations for Human Epidermal Growth Factor Receptor 2 Testing in Breast,” Journal of Clinical Oncology 31, no. 31 (2013): 3997–4013. 10.1200/JCO.2013.50.9984 24101045

[4] F. Halim , H. Agustina , Y. Azhar , and B. Hernowo , “Programmed Death Ligand‐1 Protein Expression Difference in Basal Like and Non‐Basal Like Triple Negative Breast Cancer and Its Association With Disease Free Survival and Overall Survival: A Systematic Review,” Oncology Reviews 15, no. 2 (2021): 533. 10.4081/oncol.2021.533 PMC8491008 34703525

[5] The Global Cancer Observatory , Cancer Incident in Indonesia, vol. 858 (International Agency for Research on Cancer, 2020), 1–2.

[6] B. D. Lehmann , J. A. Pietenpol , and A. R. Tan , “Triple‐Negative Breast Cancer: Molecular Subtypes and New Targets for Therapy,” American Society of Clinical Oncology Educational Book 35 (2015): e31–e39. 10.14694/EdBook_AM.2015.35.e31 25993190

[7] M. D. Burstein , A. Tsimelzon , G. M. Poage , et al., “Comprehensive Genomic Analysis Identifies Novel Subtypes and Targets of Triple‐Negative Breast Cancer,” Clinical Cancer Research 21, no. 7 (2015): 1688–1698. 10.1158/1078-0432.CCR-14-0432 PMC4362882 25208879

[8] A. Baranova , M. Krasnoselskyi , V. Starikov , et al., “Triple‐Negative Breast Cancer: Current Treatment Strategies and Factors of Negative Prognosis,” Journal of Medicine and Life 15 (2022): 153–161. 10.25122/jml-2021-0108 PMC8999097 35419095

[9] X. Wang , S. Ji , Y. Ma , et al., “Vimentin Plays an Important Role in the Promotion of Breast Cancer Cell Migration and Invasion by Leucine Aminopeptidase 3,” Cytotechnology 72, no. 5 (2020): 639–647. 10.1007/s10616-020-00402-x PMC7548013 32572729

[10] N. Yamashita , E. Tokunaga , H. Kitao , et al., “Vimentin as a Poor Prognostic Factor for Triple‐Negative Breast Cancer,” Journal of Cancer Research and Clinical Oncology 139, no. 5 (2013): 739–746. 10.1007/s00432-013-1376-6 PMC11824357 23354842

[11] G. Khalili‐Tanha and A. Shoari , “Innovative Approaches to EMT‐Related Biomarker Identification in Breast Cancer: Multi‐Omics and Machine Learning Methods,” BioTech 14, no. 3 (2025): 75. 10.3390/biotech14030075 PMC12467340 41002293

[12] H. Li , Z. He , Q. Yang , J. Wang , and Y. Ling , “Clinicopathologic Features and Prognosis of Triple‐Negative Breast Cancer in China: A Retrospective Cohort Study of Patients Under 35 Years Old,” International Journal of Clinical and Experimental Pathology 14, no. 1 (2021): 24–33. PMC7847486 33532020

[13] S. Kepuladze , G. Burkadze , and I. Kokhreidze , “Epithelial‐Mesenchymal Transition Indexes in Triple‐Negative Breast Cancer Progression and Metastases,” Cureus 16, no. 9 (2024): e68761. 10.7759/cureus.68761 PMC11456157 39371729

[14] R. Kolb and W. Zhang , “Obesity and Breast Cancer: A Case of Inflamed Adipose Tissue,” Cancers 12, no. 6 (2020): 1686. 10.3390/cancers12061686 PMC7352736 32630445

[15] A. Osborne , Q. E. S. Adnani , and B. O. Ahinkorah , “Breast Cancer Incidence in Indonesia: A Sex‐Disaggregated Analysis Using WHO Health Equity Assessment Toolkit Data,” BMC Cancer 25, no. 1 (2025): 986. 10.1186/s12885-025-14332-4 PMC12128502 40457229

[16] A. Hassan and C. Aubel , “The PI3K/Akt/mTOR Signaling Pathway in Triple‐Negative Breast Cancer: A Resistance Pathway and a Prime Target for Targeted Therapies,” Cancers 17, no. 13 (2025): 2232. 10.3390/cancers17132232 PMC12248877 40647529

[17] Y. Pan , Y. Yuan , G. Liu , and Y. Wei , “P53 and Ki‐67 as Prognostic Markers in Triple‐Negative Breast Cancer Patients,” PLoS One 12, no. 2 (2017): e0172324. 10.1371/journal.pone.0172324 PMC5325264 28235003

[18] M. I. Kokkinos , R. Wafai , M. K. Wong , D. F. Newgreen , E. W. Thompson , and M. Waltham , “Vimentin and Epithelial‐Mesenchymal Transition in Human Breast Cancer—Observations In Vitro and In Vivo,” Cells, Tissues, Organs 185, no. 1–3 (2007): 191–203. 10.1159/000101320 17587825

[19] S. Usman , N. H. Waseem , T. K. N. Nguyen , et al., “Vimentin Is at the Heart of Epithelial Mesenchymal Transition (EMT) Mediated Metastasis,” Cancers 13, no. 19 (2021): 4985. 10.3390/cancers13194985 PMC8507690 34638469

[20] L. Du , J. Li , L. Lei , et al., “High Vimentin Expression Predicts a Poor Prognosis and Progression in Colorectal Cancer: A Study With Meta‐Analysis and TCGA Database,” BioMed Research International 2018 (2018): 6387810. 10.1155/2018/6387810 PMC6000861 29955607

[21] J. Xu , Y. Chen , D. Huo , et al., “β‐Catenin Regulates c‐Myc and CDKN1A Expression in Breast Cancer Cells,” Molecular Carcinogenesis 55, no. 5 (2016): 431–439. 10.1002/mc.22292 PMC4529819 25663530

[22] M. Fedele , L. Cerchia , and G. Chiappetta , “The Epithelial‐To‐Mesenchymal Transition in Breast Cancer: Focus on Basal‐Like Carcinomas,” Cancers 9, no. 10 (2017): 134. 10.3390/cancers9100134 PMC5664073 28974015

[23] Y. Hao , D. Baker , and P. Ten Dijke , “TGF‐β‐Mediated Epithelial‐Mesenchymal Transition and Cancer Metastasis,” International Journal of Molecular Sciences 20, no. 11 (2019): 2767. 10.3390/ijms20112767 PMC6600375 31195692

[24] K. A. Won and C. Spruck , “Triple‐Negative Breast Cancer Therapy: Current and Future Perspectives (Review),” International Journal of Oncology 57, no. 6 (2020): 1245–1261. 10.3892/ijo.2020.5135 PMC7646583 33174058

